# Breast Metastases: Updates on Epidemiology and Radiologic Findings

**DOI:** 10.7759/cureus.12258

**Published:** 2020-12-24

**Authors:** Riccardo Picasso, Federico Pistoia, Federico Zaottini, Sara Sanguinetti, Massimo Calabrese, Carlo Martinoli, Lorenzo Derchi

**Affiliations:** 1 Radiology, Department of Health Sciences, University of Genova, Genoa, ITA; 2 Breast Radiology, Ospedale Policlinico San Martino, Genoa, ITA

**Keywords:** pathology, breast metastasis, secondary breast cancer, radiology, breast radiology, imaging, cancer epidemiology

## Abstract

Purpose

The aim of this study was to report the prevalence of secondary breast malignancies and analyze their radiological characteristics.

Materials and methods

We collected 42,505 pathological reports of mammary biopsies performed from January 2000 to January 2019 in our hospital database, from which we screened reports of secondary cancer of the breast. We collected and analyzed imaging data from computed tomography (CT), ultrasound (US), and mammography. Mammograms, CT scans, and US images were reviewed by two breast radiologists. Prevalence of secondary breast malignancy among suspicious breast masses and all breast malignancies were calculated.

Results

Out of 42,505 histopathology reports from mammary biopsies, we found 19,354 malignancies. We identified 33 cases of secondary breast cancers (0.08% of suspicious breast lesions, 0.17% of breast malignancies). Most common metastases were from lymphoma (23 cases, 0.05% of suspicious breast lesions, 0.12% of breast malignancies) and melanoma (six cases, 0.01% of suspicious breast lesions, 0.03% of breast malignancies). All secondary lesions were hypoechoic on US and showed high density on mammogram. On CT, 83% of the lesions appeared solid/dense, and 17% were mixed, alternating areas of iso/hyperdensity with areas of hypodensity.

Conclusion

Secondary breast cancer had a prevalence of 0.17% among all breast malignancies. No specific imaging features, characteristic of secondary breast cancer, were found.

## Introduction

In the past, breast metastases were thought to represent the 1.7%-6.6% of breast malignancies [1,2], whereas, most recently, their prevalence among mammary tumors has been updated to around 0.3%-2% [3,4]. This discrepancy may be explained considering that metastases from contralateral breast cancer, which represent the great majority of secondary breast lesions, were arbitrarily included in old series, significantly affecting their results [5,6]. Another bias could be attributed to the choice of including, or not, metastases from hematological malignancies and, in particular, secondary breast lymphoma (SBL) [7], which, when considered, resulted in the second most common secondary breast lesion [5]. Following contralateral breast cancer and lymphoproliferative disease, melanoma and lung cancer have been found to be the most common solid tumors metastasizing to the breast; metastasis from ovary, gastrointestinal tract, kidney, and sarcomas has also been reported [8,9]. The outer upper quadrant of the breast is the most frequent metastatic site, probably as a consequence of its rich vascularization [6]. A prompt distinction between non-breast tumor metastasis and breast cancer is crucial for both patients and physicians as it has been reported that the mean survival time after a diagnosis of breast metastasis is only 10 months [10]. Several attempts have been made to identify specific radiologic features able to distinguish metastases from primary breast carcinoma, although a consensus was not found among authors. The aim of this study was to review all non-breast cancer breast metastases diagnosed in our institution and to provide up-to-date data about epidemiology and radiologic features.

## Materials and methods

Our study is a single-center retrospective observational study of a consecutive series of 42,505 breast biopsies performed between January 2000 and January 2019. The histological samples were obtained mainly from core needle biopsies, but we also included fine needle, vacuum-assisted breast biopsy (VABB) and open excisional procedures. Among them, all positive reports for breast malignancies were collected. For histological purposes, neoplasms were categorized according to the World Health Organization (WHO) classification. We created two subgroups: the first of which included primary breast tumors, and the second included all secondary breast involvement from non-breast cancer metastases. Only lesions showing involvement of mammary tissue were considered as breast metastases and were not included tumors affecting the skin of the mammary region. Clinical history of patients with breast involvement from hematological malignancies was accurately screened in order to exclude primary breast lymphomas. For this purpose, following the Wiseman criteria, we did not consider patients with selective breast involvement from lymphoproliferative disease (i.e., in stage IA or IIA). Data regarding radiologic findings of secondary breast lesions were collected from our archives, when available. Metastases number and dimensions were calculated from both pathological and radiological reports. When both of them were available, we considered radiologic data: when more than one radiologic study was available, we considered the first one to detect the lesion. During images analysis, bifocal or bilateral lesions were considered separately, whereas we considered only the largest lesion in cases of diffuse/multifocal disease. Radiologic findings were collected from mammograms, computed tomography (CT) scans, and US images were reviewed by two breast radiologists. Lesions were defined as regular, irregular/spiculated, or undefined on the basis of the appearance of their margins on imaging. Regarding density, lesions were classified as solid, mixed, or colliquated/necrotizing. Epidemiological analysis on the group with breast metastases was performed, along with a collection of data about age, sex of patient, and location of the lesions. Dealing with solid tumor metastases, we analyzed if breast metastases were the first secondary lesions to be diagnosed. The ratio of breast metastases among suspicious breast lesions and breast cancer was calculated.

## Results

Epidemiology

Among 42,505 pathological reports, we identified 19,354 malignancies, including 33 cases of breast involvement by non-breast cancer metastases [0.08% of suspicious breast lesions, 95% CI 0.0008 (0.0006, 0.0011); 0.17% of breast malignancies, 95% CI 0.0017 (0.0012, 0.0024)]. All the lesions were found in women, and, at the time of the diagnosis, the mean and median ages were 65 and 69 years, respectively. With regard to the histotype, 23 lesions resulted from lymphoproliferative diseases [0.05% of suspicious breast lesions, 95% CI 0.0005 (0.0003, 0.0008); 0.12% of breast malignancies, 95% CI 0.0012 (0.0008, 0.0018)] and 10 from solid tumors (0.03% of suspicious breast lesion, 0.06% of breast malignancies). Among secondary lymphoproliferative lesions, we found 10 follicular lymphomas [43.5% of SBLs, 95% CI 0.4348 (0.2388, 0.6513); 30.3% of secondary breast lesions, 95% CI 0.303 (0.1621, 0.4887)], eight diffuse large B-cells lymphomas [DLBCL, 34.8% of SBLs, 95% CI 0.3478 (0.1719, 0.5718`; 24.2% of secondary breast lesions, 95% CI 0.2424 (0.1174, 0.4263)], two mucosa-associated lymphoid tissue (MALT) lymphomas [8.7% of SBLs, 95% CI 0.087 (0.0152, 0.2951); 6% of secondary breast lesions, 95% CI 0.0606 ´0.0106, 0.2162)], one mantle cells lymphoma [4.3% of SBLs, 95% CI 0.0435 (0.0023, 0.2397); 3% of secondary breast lesions, 95% CI 0.0303 (0.0016, 0.1751)], and one Burkitt lymphoma [4.3% of SBLs, 95% CI 0.0435 (0.0023, 0.2397); 3% of secondary breast lesion, 95% CI 0.0303 (0.0016, 0.1751)]: we were not able to find a conclusive histological diagnosis in one patient with SBLs. The median age at the diagnosis of breast metastases from hematological malignancies was 76 years. With regard to solid tumors, six cases were from melanoma [60% of solid tumor metastasis, 95% CI 0.6 (0.2737, 0.8631); 18% of secondary breast lesions, 95% CI 0.1818 (0.0762, 0.3608)], whereas we found only one case of metastasis from bladder urothelioma, one gastric adenocarcinoma (ADC), one small-cell lung cancer (SCLC), and one cervical cancer [each one representing 10% of solid tumors metastases, 95% CI 0.1 (0.0052, 0.4588); 3% of secondary breast lesions, 95% CI 0.0303 (0.0016, 0.1751)]. In six cases of solid tumor metastases (60%), breast lesions were diagnosed together with metastases involving other regions. In one case (10%) the breast was the first organ to be involved by metastases, whereas in three patients we were not able to trace the clinical history. The median age at the diagnosis of breast metastases from solid tumors was 52 years. Mean time from diagnosis of primary solid tumors and breast metastases was eight years. We found data about the side affected by lesion in 32 out of 33 patients: in 15 of them [46.8% CI 95% 0.4688 (0.2951, 0.6497)], the lesions involved the right breast; in 14 patients [43.8% CI 95% 0.4375 ´0.2684, 0.6212)], the lesions involved the left breast, whereas in three cases [9.4% CI 95% 0.0938 (0.0246, 0.2617)], the lesions resulted bilateral at the diagnosis.

Radiologic findings

**Figure 1:**
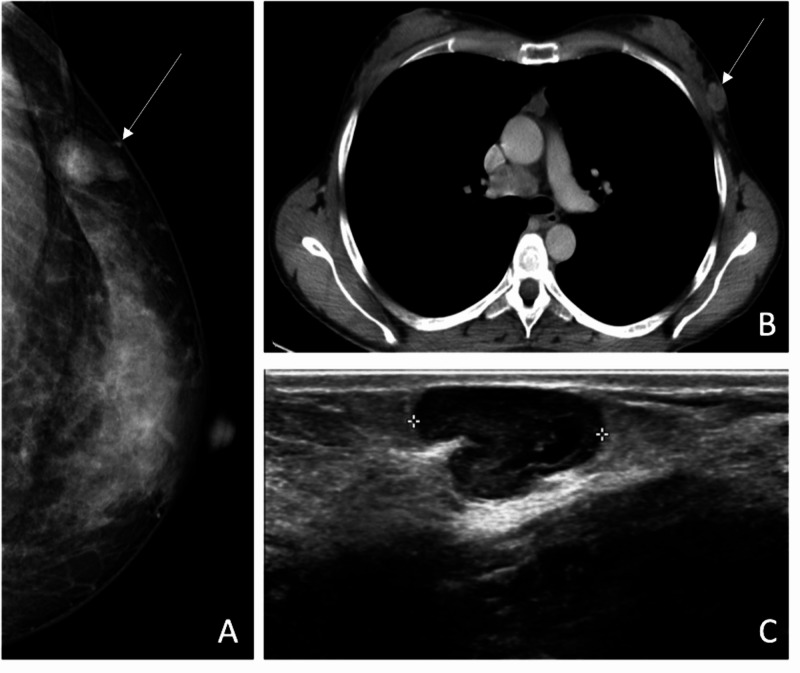
Patient with prior melanoma of the back. (A) Mammography demonstrates a round, hyperdense, circumscribed lesion, which is solid on CT scan (B). (C) Same lesion appears hypoechoic and lobulated on ultrasound.

We collected data from 20 CT, 13 US, and three mammograms. Mean lesion diameter at diagnosis was 31 mm. We analyzed a total of 24 nodules on CT. Among them, 20 [83% IC 95% 0.8333 (0.6181, 0.9452)] came up solid/dense, and four [17% IC 95% 0.1667 (0.0548, 0.3819)] appeared mixed, alternating areas of iso/hyperdensity with areas of hypodensity, reflecting necrotic and colliquate tissue. We found more heterogeneity analyzing lesion margins, with 14 nodules [58% CI 95% 0.5833 (0.3694, 0.772)] appearing smooth, six lesions [25% CI 95% 0.25 (0.106, 0.4705)] having undefined margins, and four [17% CI 95% 0.1667 (0.0548, 0.3819)] appearing spiculated/irregular. No lesion showed calcifications. Thirteen lesions were studied by means of US, and we found eight [61% CI 95% 0.6154 (0.3228, 0.8487)] regular/well-defined nodules, four [31% CI 95% 0.3077 (0.1036, 0.6112)] undefined lesions, and, in one [8% CI 95% 0.0769 (0.004, 0.3791 )] case of lymphoma, the lesion appeared as a region of ductal ectasia. All nodules were hypoechoic, showing in three cases some inhomogeneities due to anechoic/colliquated areas (Figure 1). All the lesions detected with mammography showed high density and regular margins (Table 1).

**Table 1:** All patients with secondary breast cancer from our hospital database from January 2000 to January 2019. ADC, Gastric adenocarcinoma; SCLC, small-cell lung cancer; MALT, mucosa-associated lymphoid tissue; DLBCL, diffuse large B-cell lymphomas.

	Age (y)	Side	Type	Size(mm)	CT	Ultrasound	Mammography
Case 1	56	Right	Melanoma	12	N/A	N/A	N/A
Case 2	62	Left	Melanoma	12	Solid/regular	N/A	N/A
Case 3	36	Both	Melanoma	20R-15L	Solid/regular	N/A	N/A
Case 4	69	Left	Urothelioma	60	N/A	N/A	N/A
Case 5	69	Left	Gastric ADC	14	Solid/regular	N/A	N/A
Case 6	65	Both	SCLC	14R-7L	N/A	N/A	N/A
Case 7	35	Left	Melanoma	18	Solid/regular	Regular/hypo-anechoic	Nodular/dense
Case 8	43	Right	Melanoma	17	N/A	Undefined/hypo-anechoic	N/A
Case 9	40	Right bifocal	Melanoma	18-3	N/A	N/A	N/A
Case 10	48	Left	Cervical C.	22	N/A	N/A	N/A
Case 11	76	Right	Follicular L.	70	Mixed/regular	Regular/hypoechoic	N/A
Case 12	70	Right	MALT L.	67	Solid/undefined	N/A	N/A
Case 13	88	Left bifocal	Lymphoma	20-25	Solid/regular Solid/undefined	Ductal ectasia	Nodular/dense
Case 14	88	Right	DLBCL	67	Mixed/irregular	Regular/hypoechoic	N/A
Case 15	86	Right	DLBCL	N/A	N/A	N/A	N/A
Case 16	79	Left	Follicular L.	14	Solid/regular	N/A	N/A
Case 17	82	Right	Follicular L.	N/A	Solid/regular	Regular/hypoechoic	N/A
Case 18	51	Left	Follicular L.	10	Solid/regular	N/A	N/A
Case 19	83	Left	DLBCL	80	Mixed/undefined	N/A	N/A
Case 20	70	Right	DLBCL	N/A	Solid/regular	N/A	N/A
Case 21	30	Both	Burkitt L.	63R-35L	Solid/undefined	Regular/hypoechoic	N/A
Case 22	78	Left	Follicular L.	15	Solid/regular	N/A	Nodular/dense
Case 23	47	Left	DLBCL	26	Solid/regular	Regular/hypoechoic	N/A
Case 24	70	Right	Follicular L.	19	Solid/irregular	Regular/hypoechoic	N/A
Case 25	86	Right	Mantle cells L.	156	Mixed/undefined	N/A	N/A
Case 26	79	Right	Follicular L.	28	Solid/regular	N/A	N/A
Case 27	77	Left	Mantle cells L.	19-24	Solid/irregular	Undefined/hypoechoic	N/A
Case 28	70	Right bifocal	DLBCL	N/A	N/A	Undefined/hypo-anechoic	N/A
Case 29	61	Left	DLBCL	N/A	N/A	N/A	N/A
Case 30	57	Left	DLBCL	N/A	N/A	N/A	N/A
Case 31	77	Right	Follicular L.	8	N/A	Undefined/hypoechoic	N/A
Case 32	69	N/A	Follicular L.	N/A	N/A	N/A	N/A
Case 33	58	Right	Follicular L.	12	N/A	Regular/hypo-anechoic	N/A

## Discussion

Relying on histological diagnosis in clinical studies, the frequency of breast metastases from extramammary malignancy compared with breast carcinoma varies between 0.2% and 1.3% [11], with higher frequencies (2%-7%) in post-mortem studies [2]. In our series, breast metastases represent 0.08% of suspicious breast lesions and 0.17% of breast malignancies. This variability can be explained by different inclusion criteria. According to Georgiannos et al., when metastases from contralateral breast cancer are included, the frequency of secondary breast tumor is set at 3% among breast malignancies, while it represents 0.43% when only non-breast cancer metastases are considered [5]. We have applied this latter restriction since the differentiation between synchronous primary breast tumor, and metastases from cancer in the contralateral breast are achieved only by means of massive sequencing assay, not routinely performed. We also paid attention to differentiate between primary and SBLs using the Wiseman clinical criteria.

Considering solid tumor metastases, in 60% of cases, breast lesions were diagnosed together with secondarism to other organs. In only one case (10%), a breast lesion was the first metastasis to be diagnosed, and the imaging protocol for breast palpable lesion was followed. Mean time from diagnosis of primary solid tumor and breast metastasis was eight years. In literature, the time from initial diagnosis to metastasis to the breast ranges between one month and 15 years, with significant variations due to geographical and/or racial biases [12]. A long interval is well recognized for some tumor types such as malignant melanoma and ovarian carcinoma and is more common in Western population [12]. Unilateral or bilateral breast metastatic involvement at time of diagnosis (90.6% and 9.4%, respectively) showed frequency in accordance with data found in literature [13,14]. Among the lesions included in our study, 0.05% of suspicious breast lesions and 0.12% of breast malignancies resulted from secondary involvement in patients with systemic lymphoproliferative diseases, while 0.03% of suspicious breast lesions and 0.06% of breast malignancies were metastases from solid tumors. Regarding this latter group, the great majority of lesions resulted in metastatic melanoma (six cases, 60% of solid tumor metastases, 18% of secondary breast lesions). Melanoma is the most frequent type of breast metastasis from solid tumor. Following data from the Caucasian population and, as observable in Table 1, melanoma is the commonest type of metastasis among younger patients. Hematologic malignancies represent, overall, the most common type of breast secondarism. As observed by Georgiannos et al., the presence of hematologic malignancies in the breast are becoming relatively more frequent when compared to the first part of our century [5]. This trend is confirmed in our study, which reports a higher frequency (70%) of hematologic malignancies compared to Georgiannos et al.’s work (53%). We did not include patients of pediatric age as the cases analyzed had been retrieved from archives in an adult hospital, missing some histotypes of metastases typical of younger age as rhabdomiosarcoma [15] and acute lymphoblastic leukemia. The vast majority of secondary neoplasms in the published literature were encountered in women (95%). In our series, no males have been included. It is likely that the differences in breast size and vascularity between the two groups play a role, but it is possible that there are other reasons for this gender bias, such as hormonal factors. As a matter of fact, a high occurrence of breast metastases is reported in pubescent girls, women in pregnancy or the lactating state, and in men with prostatic cancer undergoing hormone replacement therapy [16]. It has been suggested that estrogens may increase the breast stroma vascularity, representing a predisposing factor for the development of metastases [15]. Most of the secondary involvement of the breast occurred in older patients, with a mean age at diagnosis of 65 years. Vergier et al. found a mean age of 41 with a similar distribution of histotypes [17]: the authors explained the relatively young age of the affected patients as the result of a greater vascularity of breast tissue in younger age and a lower fibrotic tissue component. Although this consideration is consistent with the physiopathology of metastasizing mechanism, demographic age distribution in a determined region may play a significant role.

US is integral in the evaluation of all palpable breast masses and plays a key role in the diagnosis of metastatic lesions presenting as palpable lumps. All our cases studied with US presented features congruent with literature, in particular the absence of distortion due to lack of desmoplastic reaction, the presence of pseudo-cystic areas due to intra-tumoral necrotic process, and the absence of tumoral calcification. Axillary lymph node involvement was less common in metastases than in primary breast cancers [3]. On CT the majority of lesions appeared solid-dense; 17% of lesions presented a heterogenous density reflecting necrosis and corresponding to the pseudo-cystic areas detected with ultrasound. On mammogram, lesions have been more often described as dense and rounded, not causing distortion of the surrounding parenchyma, neither showing calcifications [18]; lesions from ovarian, thyroid, or mucin-producing gastrointestinal tract carcinoma may appear ill-defined, with parenchymal distortion and sporadic calcifications [19]. The low number of mammograms in our series is due to the fact that most of the lesions were identified in the context of CT follow-up in patients with a previous history of neoplasm; another reason is that some patients were submitted to ultrasound guided biopsy at our hospital after a previous positive mammography in another center. Our findings were consistent with the ones displayed by the others imaging methods: the lack of significant desmoplastic response near the lesion explained the less frequent spiculations found on the mammogram [18] (17%) and is related to the sharp transition at the border of the lesion found on histologic examination [13]. On US, metastases have been most commonly described as hypoechoic [20,21], but hyperechoic lesions from lymphoproliferative disease have also been reported [22]. In one paper, the US appearance of breast metastases was related to the way through which tumors spread to this region; hematogenous metastases were reported to appear well-defined with regular margins, whereas lymphatic metastases were linked to ill-defined lesions associated with desmoplastic reaction of the surrounding parenchyma [23]. MR in the correct clinical setting could be more specific in diagnosing metastases from melanoma due to their high content of melanin and consequent high signal on T1-weighted sequences [24]. However, in most cases, MR findings are misleading; Surov et al. found that breast metastases on MR can resemble benign lesions like fibroadenoma, whereas their kinematic enhancing curves are in most cases identical to that of primary breast cancer [19]. Our study has some limitations. In a total of 36 imaging studies analyzed, CT accounted for 55%, whereas conventional techniques accounted for only 45%, of which only 8.3% of cases were studied by mammography; these data should be considered since CT has low spatial resolution, and therefore it is not part of the conventional workup for breast calcifications [25].

## Conclusions

In our series, secondary breast cancer has a prevalence of 0.17% among all breast malignancies; the primary tumors from which breast metastases arise more frequently are lymphoma and melanoma. We did not find any specific imaging feature able to provide a definite diagnosis of breast metastases without histological sampling. For this purpose, we believe that advanced imaging techniques like functional sequences on MR may help in the future to better characterize indeterminate breast lesion in specific cohorts of oncologic patients.
